# Supplementary material for: Genome-Wide Identification and Expression Analysis of Homeodomain Leucine Zipper Subfamily IV (HD-ZIP IV) Gene Family in Cannabis sativa L
Source: Plants (Basel). 2022 May 13;11(10):1307. doi: 10.3390/plants11101307 (PMC9144208; doi:10.3390/plants11101307)
Supplement: Supplementary file 1 [file plants-11-01307-s001.zip › plants-1659373-supplementary.pdf]

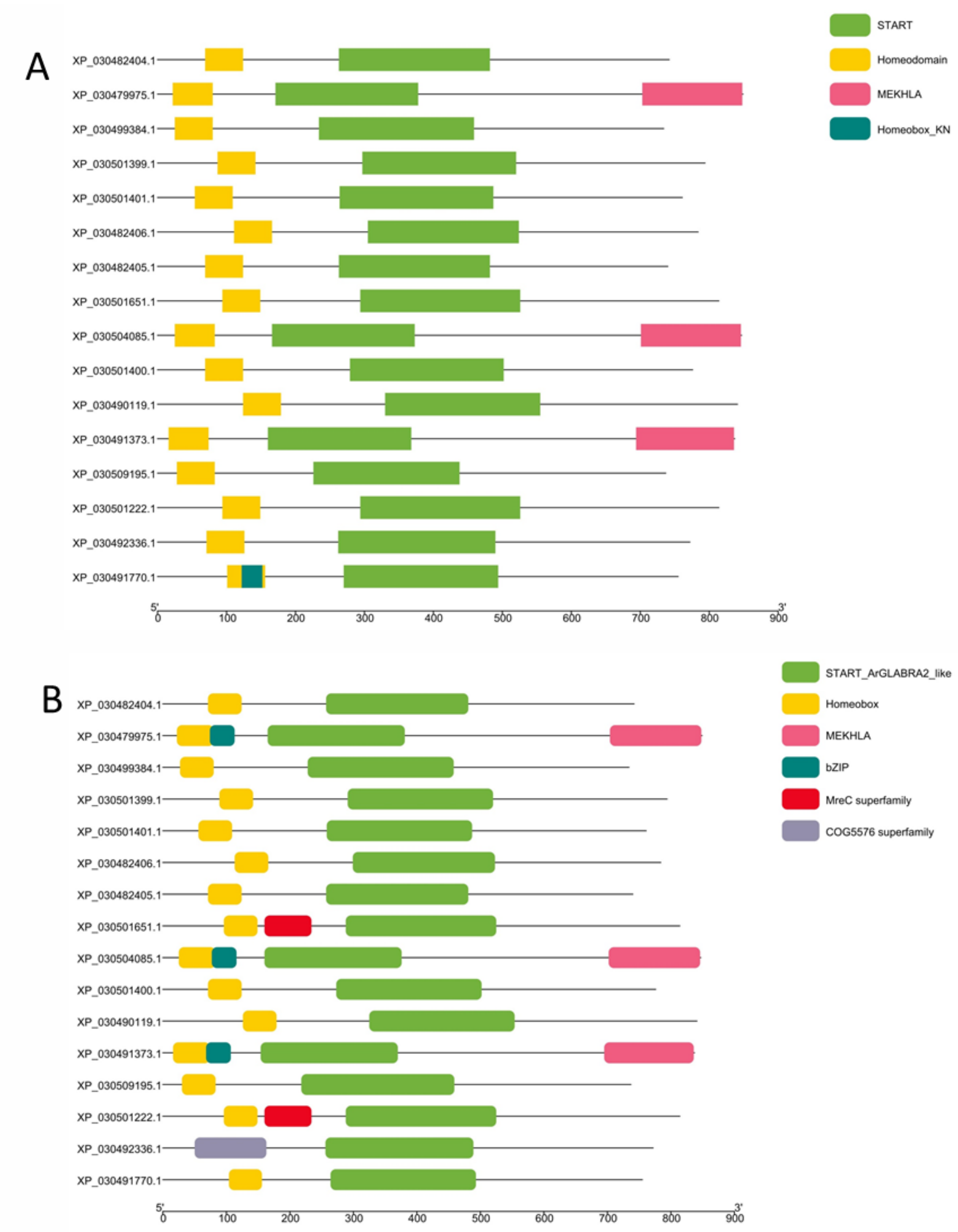

Figure S1. Schematic representation of conserved motifs in Cannabis HDZ IV proteins by Pfam database (A) and Conserved Domain Database (B),

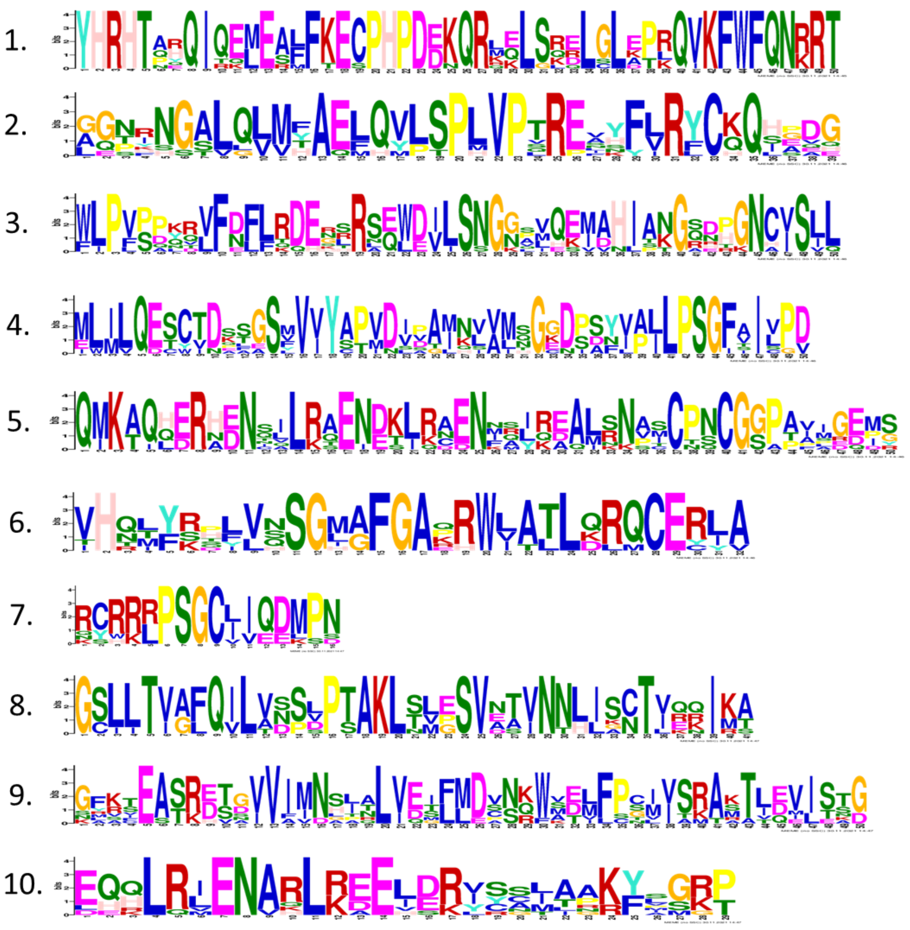

Figure S2. Sequence logos corresponding to the conserved motifs present over cannabis HDZ IV proteins.

Table S1. Primers

Gene name	qPCR forward primer	qPCR reverse primer
HDG5	ACTGCATGACTCCGAGATTTAG	GTGCCTAGAGTAGATGCTGATG
ANTHOCYANINLESS 2 isoform X1	TTACCGGCCATTATTGAGTTCC	GTTATTCCTGCAGTGTGGTC
GLABRA 2	CGGTATGAATAATGGTCCTAAGAG	TCCTTCCATTGATTCACATCCA
HDG11	GTTCTTCGAGCTTTCAACACC	GGCAGTACACCACCATTGAC
ANTHOCYANINLESS 2 isoform X2	TGGCTATTCCAAGGTTACGTG	CAAGTACTCGCATTGTCTTTGG
HDG5-like	CTTCAATATCAACCTTCACTTCCC	TTCAACCCATGTGACACTTGAG
HDG2	GGGCAGTTGTTGATGTTTCTC	CCATGTCACCTTAGAGTATCCA
ROC8-like	AATCAGAGTGTCAGTTCGTAAGG	AGAACATCCCACTGGAGTCTG
PROTODERMAL FACTOR 2	TATAGCCAATGGGCGTGAT	CAACTGGTGCATAAATCACA
Ubiquitin	GCCAGGATGGCAATGAAGTA	GCTATAGAGTTGAACTCCACAG

Table S2. Putative targets of HDZ IV transcription factors.

Gene	Number of 5′-GCATTAAATGC-3′motif	Gene Annotation
XM_030622497.1	1	PREDICTED: Cannabis sativa proline-rich protein 36 (LOC115695434), mRNA
XM_030622779.1	1	PREDICTED: Cannabis sativa uncharacterized LOC115695708 (LOC115695708), mRNA
XM_030623013.1	1	PREDICTED: Cannabis sativa transcription termination factor MTEF18, mitochondrial (LOC115695917), mRNA
XM_030623245.1	1	PREDICTED: Cannabis sativa uncharacterized LOC115696341 (LOC115696341), mRNA
XM_030623347.1	1	PREDICTED: Cannabis sativa uncharacterized LOC115696446 (LOC115696446), mRNA
XM_030623509.1	1	PREDICTED: Cannabis sativa uncharacterized LOC115696614 (LOC115696614), mRNA
XM_030623927.1	1	PREDICTED: Cannabis sativa AMP deaminase (LOC115697031), transcript variant X2, mRNA
XM_030624933.1	1	PREDICTED: Cannabis sativa DNA replication complex GINS protein SLD5 (LOC115697802), mRNA
XM_030624994.1	1	PREDICTED: Cannabis sativa farnesylcysteine lyase (LOC115697848), mRNA

XM_030625176.1	1	PREDICTED: Cannabis sativa putative F-box/LRR-repeat protein 23 (LOC115698001), mRNA
XM_030625390.1	1	PREDICTED: Cannabis sativa U-box domain-containing protein 1 (LOC115698200), mRNA
XM_030625492.1	1	PREDICTED: Cannabis sativa gamma-interferon-responsive lysosomal thiol protein (LOC115698280), mRNA
XM_030625813.1	1	PREDICTED: Cannabis sativa cellulose synthase-like protein H1 (LOC115698677), transcript variant X1, mRNA
XM_030626179.1	1	PREDICTED: Cannabis sativa F-box protein SNE (LOC115698964), mRNA
XM_030626921.1	1	PREDICTED: Cannabis sativa putative DNA (cytosine-5)-methyltransferase CMT1 (LOC115699482), mRNA
XM_030627406.1	1	PREDICTED: Cannabis sativa uncharacterized LOC115699863 (LOC115699863), mRNA
XM_030628894.1	1	PREDICTED: Cannabis sativa tubulin-folding cofactor B (LOC115701175), mRNA
XM_030629883.1	1	PREDICTED: Cannabis sativa nodulation-signaling pathway 2 protein (LOC115702418), mRNA

XM_030630414.1	1	PREDICTED: Cannabis sativa protein UPSTREAM OF FLC (LOC115702942), transcript variant X1, mRNA
XM_030631028.1	1	PREDICTED: Cannabis sativa O-fucosyltransferase 28-like (LOC115703794), mRNA
XM_030631124.1	1	PREDICTED: Cannabis sativa uncharacterized LOC115703887 (LOC115703887), mRNA
XM_030631839.1	1	PREDICTED: Cannabis sativa wall-associated receptor kinase-like 3 (LOC115704637), mRNA
XM_030632507.1	1	PREDICTED: Cannabis sativa uncharacterized LOC115705235 (LOC115705235), transcript variant X1, mRNA
XM_030633407.1	1	PREDICTED: Cannabis sativa peptidyl-tRNA hydrolase 2, mitochondrial (LOC115705927), mRNA
XM_030633517.1	2	PREDICTED: Cannabis sativa G-protein coupled receptor 1 (LOC115706017), mRNA
XM_030633774.1	1	PREDICTED: Cannabis sativa uncharacterized LOC115706204 (LOC115706204), mRNA
XM_030634242.1	1	PREDICTED: Cannabis sativa uncharacterized LOC115706553 (LOC115706553), mRNA

XM_030634601.1	1	PREDICTED: Cannabis sativa cytochrome P450 711A1 (LOC115706844), mRNA
XM_030635119.1	1	PREDICTED: Cannabis sativa MADS-box protein FBP24-like (LOC115707234), transcript variant X1, mRNA
XM_030636562.1	1	PREDICTED: Cannabis sativa probable glycosyltransferase At3g07620 (LOC115708322), transcript variant X1, mRNA
XM_030637341.1	1	PREDICTED: Cannabis sativa DNAJ protein JJJ1 homolog (LOC115709278), transcript variant X1, mRNA
XM_030637534.1	1	PREDICTED: Cannabis sativa transcription factor BIM1 (LOC115709430), transcript variant X1, mRNA
XM_030637765.1	1	PREDICTED: Cannabis sativa protein QUIRKY (LOC115709623), mRNA
XM_030638081.1	1	PREDICTED: Cannabis sativa probable inactive receptor kinase At5g67200 (LOC115709841), mRNA
XM_030638567.1	1	PREDICTED: Cannabis sativa WD repeat and HMG-box DNA-binding protein 1 (LOC115710221), mRNA

XM_030639218.1	1	PREDICTED: Cannabis sativa dynamin-related protein 1E (LOC115710860), mRNA
XM_030639782.1	1	PREDICTED: Cannabis sativa alpha carbonic anhydrase 7-like (LOC115711440), mRNA
XM_030639993.1	1	PREDICTED: Cannabis sativa carotenoid 9,10(9',10')-cleavage dioxygenase 1 (LOC115711621), transcript variant X1, mRNA
XM_030640064.1	1	PREDICTED: Cannabis sativa ankyrin repeat domain-containing protein 1 (LOC115711685), mRNA
XM_030640451.1	1	PREDICTED: Cannabis sativa uncharacterized LOC115712201 (LOC115712201), mRNA
XM_030641975.1	1	PREDICTED: Cannabis sativa uncharacterized LOC115713490 (LOC115713490), mRNA
XM_030642294.1	1	PREDICTED: Cannabis sativa U-box domain-containing protein 15 (LOC115713809), mRNA
XM_030643475.1	1	PREDICTED: Cannabis sativa transcription factor bHLH63 (LOC115714717), transcript variant X1, mRNA
XM_030643671.1	1	PREDICTED: Cannabis sativa glutathione S-transferase U17 (LOC115714913), mRNA

XM_030644486.1	1	PREDICTED: Cannabis sativa zinc finger protein ZAT4 (LOC115715815), mRNA
XM_030645201.1	3	PREDICTED: Cannabis sativa uncharacterized protein At3g49140 (LOC115716414), mRNA
XM_030645594.1	1	PREDICTED: Cannabis sativa uncharacterized LOC115716718 (LOC115716718), mRNA
XM_030646233.1	1	PREDICTED: Cannabis sativa V-type proton ATPase subunit F (LOC115717265), mRNA
XM_030647270.1	1	PREDICTED: Cannabis sativa pentatricopeptide repeat-containing protein At1g53600, mitochondrial (LOC115718481), transcript variant X1, mRNA
XM_030647841.1	1	PREDICTED: Cannabis sativa regulator of nonsense transcripts UPF3 (LOC115718999), transcript variant X1, mRNA
XM_030648752.1	1	PREDICTED: Cannabis sativa DDT domain-containing protein PTM (LOC115719637), transcript variant X3, mRNA
XM_030649189.1	1	PREDICTED: Cannabis sativa zinc finger BED domain-containing protein RICESLEEPER 2-like (LOC115720022), mRNA

XM_030649300.1	1	PREDICTED: Cannabis sativa uncharacterized LOC115720141 (LOC115720141), mRNA
XM_030649628.1	1	PREDICTED: Cannabis sativa cytochrome P450 714C2 (LOC115720479), mRNA
XM_030649781.1	1	PREDICTED: Cannabis sativa heavy metal-associated isoprenylated plant protein 35 (LOC115720619), transcript variant X1, mRNA
XM_030650042.1	1	PREDICTED: Cannabis sativa respiratory burst oxidase homolog protein B (LOC115720858), mRNA
XM_030650068.1	1	PREDICTED: Cannabis sativa uncharacterized LOC115720870 (LOC115720870), mRNA
XM_030653123.1	1	PREDICTED: Cannabis sativa uncharacterized LOC115723638 (LOC115723638), mRNA
XM_030654934.1	2	PREDICTED: Cannabis sativa uncharacterized LOC115725414 (LOC115725414), mRNA
